# Evaluating the feasibility of a rehabilitation intervention including physical activity as structured active play for preschoolers diagnosed with cancer during the first 6 months of treatment—a study based on data from the RePlay trial

**DOI:** 10.1007/s00431-025-06350-y

**Published:** 2025-08-06

**Authors:** Anna Pouplier, Martin Kaj Fridh, Peter Schmidt-Andersen, Anne Meilandt Langdal Nielsen, Helle Winther, Jan Christensen, Hanne Bækgaard Larsen

**Affiliations:** 1 Department of Pediatric and Adolescent Medicine, The Juliane Marie Centre, Copenhagen University Hospital - Rigshospitalet, Blegdamsvej 9, DK-2100 Copenhagen, Denmark; 2 Department of Clinical Medicine, Faculty of Health and Medical Sciences, University of Copenhagen, Copenhagen, Denmark; 3 Department of Occupational Therapy and Physiotherapy, Center of Head and Orthopedic Surgery, Copenhagen University Hospital - Rigshospitalet, Copenhagen, Denmark; 4 Department of Nutrition, Exercise and Sports, Faculty of Science, University of Copenhagen, Copenhagen, Denmark

**Keywords:** Preschoolers, Cancer, Rehabilitation, Physical activity, Structured active play, Feasibility

## Abstract

The aim of this study was to evaluate the feasibility of a rehabilitation intervention consisting of structured active play designed for preschoolers diagnosed with cancer and their parents during treatment. Eighty-four consecutively enrolled children in RePlay (NCT04672681) were included. Feasibility was addressed across five domains: (a) participation acceptance, (b) attrition during the intervention period, (c) completion of outcome assessments, (d) adherence to the intervention, and (e) occurrence of adverse events during the intervention or outcome assessments. Further, survey data was used to describe the adherence practicalities. Descriptive statistics were used for data analysis. We found an 84% acceptance and 4% attrition rates. Completion rates of the primary outcome of gross motor function assessed with the Peabody Developmental Motor Scales – Second Edition (PDMS-2) were 44% at baseline and 80% at end-of-intervention. Median adherence to the intervention during admission was 57% [IQR 50 to 68%]. No serious adverse events were reported. Parents’ survey responses showed that 94% considered the intervention relevant or very relevant for themselves and their child, though 54% found the scheduled timing of the hospital-based intervention unsuitable.

*Conclusion*: It is feasible to conduct a rehabilitation intervention consisting of structured active play for preschoolers during intensive cancer treatment. Assessment of gross motor function posed certain challenges, but completing evaluations at the end of the intervention was feasible. These findings emphasize the importance of flexible interventions and considerations of the timing and duration of outcome assessment. Future studies should incorporate strategies to enhance adherence and minimize participation barriers.

**Table:** 

**What is Known:** • *Children diagnosed with cancer experience treatment-related physical side effects.* • *Research shows promising effects and feasibility of physical activity interventions for school-aged children diagnosed with cancer; however, knowledge on interventions specifically for preschoolers with cancer is scarce.*
**What is New:** • *It is feasible to conduct a rehabilitation intervention consisting of structured active play for preschoolers during intensive cancer treatment, including assessment of gross motor function.* • *Consideration of timing and duration of the outcome assessment and a flexible intervention may enhance adherence and minimize participation barriers.*

**Supplementary Information:**

The online version contains supplementary material available at 10.1007/s00431-025-06350-y.

## Introduction

Children undergoing cancer treatment face significant physical challenges due to the combination of chemotherapy, radiotherapy, glucocorticoids, and sedentary routines [1]. These treatments can affect their central and peripheral nervous systems, as well as muscle morphology [1–3], leading to impaired cardiorespiratory fitness, muscle strength, and gross motor functions [4–13] and reduced physical activity [14]. Common side effects include fatigue, sleep disruption, and nausea, which further decrease their energy and motivation for physical activities [15]. Little is known about the specific physical impairments of preschoolers; however, one study indicates that preschool children (6 months to 5 years old) experience severe walking difficulties and loss of ability to support themselves on their legs [16], and another study shows that gross motor function impairments can persist up to 2 years after treatment, affecting the children’s balance and more complex motor skills as they grow [17]. Impairments in gross motor functions can be particularly critical for preschool children, as gross motor function development is important for physical activity in early childhood [18, 19]. Simultaneously, being physically active contributes to improved and better development of gross motor functions [20, 21], which is associated with other developmental areas such as cognitive, social, and personal development [22]. Research shows that physical activity interventions including endurance, strength, and/or stretch exercises during cancer treatment are feasible for children aged 1–18 years with significant effects on cardiorespiratory fitness, physical function, and muscle strength [23–33], but no to little effect on motor performance [13, 34–36]. All of the previous studies include a broad age range with no distinction between results for the different age groups; consequently, no evidence exists on the feasibility or effect of interventions specifically for preschool children, who account for roughly 50% of children diagnosed with cancer [37, 38]. Preschool children are intrinsically motivated to play, and play is how they are physically active [39, 40]. Therefore, rehabilitation interventions for preschoolers diagnosed with cancer must be designed to encompass their age and developmental stage, and finding feasible rehabilitation approaches suitable for this age group of children is needed. This study aimed to evaluate the feasibility of a novel rehabilitation intervention consisting of structured active play [41] designed specifically for preschoolers during cancer treatment.

## Methods

This study is reported according to the CONSORT (CONsolidated Standards Of Reporting Trials) checklist for pilot and feasibility trials [42].

### The RePlay trial

This study reports outcomes of feasibility within the two-armed superiority randomized controlled trial—Rehabilitation including structured active play for preschoolers with cancer (RePlay) [41]. A detailed description, including rationale, of the structured active play intervention—referred to as the RePlay Model—is published elsewhere [43]. Data collection of the RePlay trial is still ongoing (NCT04672681).

#### Setting

Participants were recruited from the largest pediatric oncology center in Denmark at the Department of Pediatric and Adolescent Medicine at Copenhagen University Hospital, Rigshospitalet.

#### Participants and intervention

For this feasibility study, we included data from the total sample size of all 84 enrolled children in the RePlay trial. Children were included in the RePlay trial if they were newly diagnosed with cancer, 1–5 years old, treated with chemotherapy and/or radiation, and had parents who could communicate in Danish. Children were enrolled using a consecutive sampling method until the sample size was reached. Upon inclusion and baseline physical assessment, children were randomized to the intervention or usual care group stratified by age (< 36 months or ≥ 36 months) due to age-specific development and diagnosis (hematological cancers, tumors in the central nervous system, or extracranial solid tumors). A detailed description of the sample size, inclusion and exclusion criteria, randomization, and the intervention can be found in the study protocol [41].

The intervention was a combined hospital- and home-based program of daily structured active play. The hospital-based structured active play was offered as 45-min group sessions for the children and their parents when they were at the hospital. Respecting age-specific development, children were divided into two groups for the group sessions: < 36 months and ≥ 36 months. The sessions were offered thrice weekly (Tuesday, Thursday, and Friday) at scheduled times in the afternoon (e.g., < 36 months at 1:00–1:45 pm and ≥ 36 months at 2:00–2.45 pm) and conducted outside the pediatric ward in the centrally located pediatric physiotherapy by healthcare professionals (i.e., exercise professionals or a physiotherapist). All facilitating healthcare professionals followed the RePlay model for each session, which ensures a systematic and consistent structure of the sessions and aims to challenge the child motorically within the zone of proximal development, while still accommodating the children’s fluctuating daily well-being and different preferences in play [43]. On the days of the group sessions, all families in the intervention group would receive a reminder via text message, and the facilitating healthcare professional would also contact the admitted families face-to-face. If the child was unable to leave the ward for the group sessions (e.g., due to treatment procedures or isolation), the child was offered a session in the child’s patient room, structured as the group sessions and facilitated by the healthcare professional. For the home-based part of the intervention, parents were encouraged to facilitate individual active play with their child daily and register what they did in a logbook. The parents were given inspirational material with different play activities appropriate for their child’s age. The material is described in more detail in the protocol [41]. Participants allocated to the usual care group received standard care of treatment, including physical therapy if needed. The parents received no instructions or supervision for home-based active play.

#### Outcome measures for the RePlay randomized controlled trial

Outcome assessments were conducted at the following time points: baseline within 14 days of treatment initiation; mid-intervention at 3 months after treatment initiation (+/− 14 days); end-of-intervention (primary endpoint) at 6 months after treatment initiation (+/− 14 days) [41].

Outcome assessment included gross motor function measured using the gross motor function subtest of the Peabody Developmental Motor Scales, Second Edition (PDMS-2), which contains three domains (i.e., stationary (30 items), locomotion (89 items), and object manipulation (24 items)) [44], physical capacity measured using the 6-min walk test [45, 46], handgrip strength measured with the KLS Martin Vigorimeter (KLS Martin group, 78532 Tuttlingen, Germany) [47, 48], and level of daily function measured through the Pediatric Evaluation of Disability Inventory (PEDI) [49]. The study protocol describes all outcome measures in detail [41].

### Evaluating feasibility

Feasibility was evaluated by acceptance, attrition, completion, adherence, adverse events, and an intervention evaluation survey. The feasibility outcomes were inspired and modified from Bowen et al. [50] and the feasibility studies by Schmidt-Andersen et al. [51] and Hubbard et al. [52].

#### Acceptance

Acceptance was defined as the percentage of eligible participants who entered the trial [51]. To assess the acceptability, we registered the number of eligible children and children included in the trial upon their parent’s consent. If the family provided a reason for not participating, this was registered.

#### Attrition

The attrition rate was defined as the percentage of participants who dropped out of the trial before the primary endpoint (6 months after treatment initiation) [51]. We further registered the reasons for dropping out of the trial.

#### Assessment completion

Assessment completion was defined as the percentage of participants completing outcome assessment at each timepoint [52]. The three outcomes for physical testing (PDMS-2, 6-min walk test, and handgrip strength) of the children were scheduled together in one test assessment, where the priority was to complete the primary outcome of the gross motor function subtest of the PDMS-2. Thus, the primary completion rate reported is the percentage of sessions where the three domains of the gross motor function subtest of the PDMS-2 were completed.

The PEDI assessment, requiring active parental participation, was preferably conducted during the child’s physical assessment or, if needed, scheduled separately within the same week.

Secondary completion rates included the number of scheduled assessments—indicating the parent’s acceptance of the outcome measures as well as the possibility for a test assessment to be scheduled; the number of assessments where the entire test battery (i.e., PDMS-2, 6-min walk test, and handgrip strength) was completed—indicating whether the full test battery was completed; scheduling and completion of the PEDI. Reasons for non-completion of the outcomes were reported.

#### Adherence to intervention

Adherence to the intervention was defined as participation in the hospital-based part of the intervention [52]. This was reported in two ways: (a) the median number of hospital-based healthcare professional-led sessions that the child participated in when admitted to the hospital during the first 6 months of cancer treatment; (b) the median of the total number of hospital-based healthcare professional-led sessions that the child participated in, both when admitted to the hospital and when visiting the outpatient clinic; (c) the mean number of hospital-based healthcare professional-led sessions per week during 6 months. We registered reasons for non-participation in structured active play sessions.

#### Adverse events

Adverse events were defined and graded using the National Cancer Institute’s common criteria for mild, moderate, and severe adverse events [53]. All adverse events experienced during the hospital-based structured active play sessions or outcome assessments were registered.

#### Parent evaluation survey to assess feasibility

All parents of children in the intervention group answered a short intervention evaluation survey within 2 weeks after end-of-intervention. The choice to embed an evaluation survey was made to provide a broader perspective of the intervention, including feasibility. For this study, the relevance of the intervention for the parents and their child, time of day and location of the hospital-based part of the intervention, and length of the intervention were evaluated. The complete evaluation survey can be found in Supplemental file 1 S1).

### Statistical analysis

Descriptive statistics were used to summarize outcomes related to acceptance, attrition, completion, adherence, adverse events, and the parent evaluation survey. Acceptance, attrition, scheduling, completion, and reasons for non-scheduling, non-completion, and non-participation were reported as frequencies, percentages, and rates. Subgroup analyses were conducted for outcome completion and presented as frequencies and percentages, stratified by sex, age group (i.e., < 36 months vs. ≥ 36 months at inclusion), diagnostic category (i.e., hematological cancers, extracranial solid tumors, and central nervous system tumors), and trial group (intervention vs. usual care). For the intervention group, adherence was reported at the individual level as frequencies and percentages and at the group level as median and interquartile range (IQR) for overall adherence and adherence to hospital-based sessions. Individual adherence for each child in the intervention group was visualized using a bar chart. Adverse events were reported and presented separately for the intervention and usual care groups and categorized as either adverse events or serious adverse events. Items in the parent evaluation survey were scored on a 3- or 5-point Likert scale or yes or no answers. The 5-point Likert scale answers were divided into three groups depending on the wording of the responses (e.g., very good/good, neither/nor, or bad/very bad). All items were reported separately and as frequencies and percentages.

## Results

### Acceptance

**Fig. 1:**
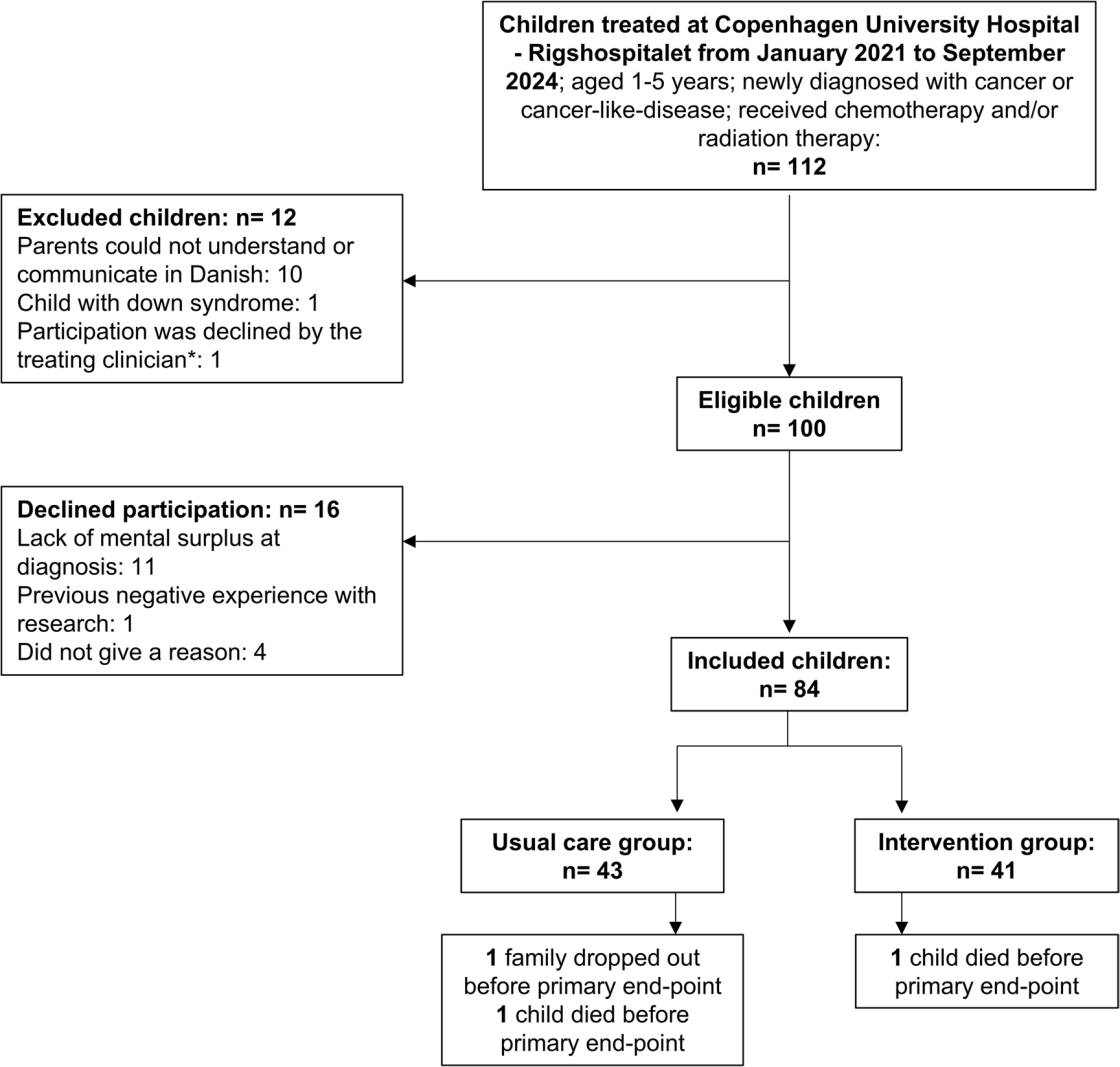
Flowchart of the inclusion in the RePlay trial. *It is not part of our inclusion or exclusion criteria that the treating clinician was to screen the children for physical eligibility. However, one child was excluded by the treating physician due to a major traumatic injury caused by the cancer before treatment initiation

From January 2021 to September 2024, 84 of 100 eligible children (84%) accepted to participate in the trial. A flowchart including reasons for declining participation is presented in Fig. 1.

#### Characteristics of included participants

Table 1 shows the participant characteristics at inclusion of the 84 children enrolled in the RePlay trial.

**Table 1:** Participants characteristics, at inclusion, of the 84 children enrolled in the RePlay trial

Characteristics at inclusion	All; *n* = 84	Intervention group; *n* = 41	Usual care group; *n* = 43
Age < 36 months	33	17	16
Age ≥ 36 months	51	24	27
Female	42	23	19
Male	42	18	24
Hematological cancers	55	27	28
Extracranial solid tumors	16	8	8
Central nervous system tumors	13	6	7

### Attrition

One family allocated to the usual care group dropped out of the study before end-of-treatment assessment due to a lack of perceived relevance, and two children died during the primary study period (3/84 = 4%, attrition).

### Completion

Table 2 shows an overview of the scheduled and conducted assessments. Reasons for the low completion rate of the full PDMS-2 at baseline were the children’s willingness to participate due to temper/motivation/concentration or pain/physical deficiencies, which were the reasons for non-completion. A full overview of the reasons for non-scheduling and non-completion can be found in Tables 3 and 4. The outcome completion rates for the sub-groups can be found in Supplemental file 2 (Table S1).

**Table 2:** Overview of scheduling and outcome completion at the different time points

	Baseline (treatment initiation) *n* = 84	Mid-intervention (3 months) *n* = 83*	End-of-intervention (6 months) *n* = 81**
Scheduled test assessment	67 (80%)	63 (76%)	73 (90%)
Completion of the PDMS-2^1^			
• Fully completed	37 (44%)	55 (66%)	65 (80%)
• Completion of at least one domain in the subtest	15 (18%)	7 (8%)	5 (6%)
• Not completed	15 (18%)	1 (1%)	3 (4%)
Scheduled PEDI^2^-sessions	83 (99%)	70 (84%)	77 (95%)
Completion of the PEDI	83 (99%)	70 (84%)	77 (95%)
Completion of the 6-min walk test	7 (8%)	18 (22%)	24 (30%)
Completion of the handgrip strength	23 (27%)	34 (41%)	44 (54%)
Completion of the full test-battery (i.e., PDMS-2, 6-min walk test, and handgrip strength) at the test-sessions	4 (5%)	13 (16%)	22 (27%)

*One child died before end-of-intervention

**Two children died, and one child dropped out before end-of-intervention

^1^Peabody Developmental Motor Scale – Second Edition

^2^Pediatric Evaluation of Disability Inventory

**Table 3:** Reasons for non-scheduling of the test assessments (i.e., PDMS-2, 6-min walk test, and handgrip strength) and the PEDI assessments at baseline (treatment initiation), mid-intervention (3 months), and end-of-intervention (6 months)

	**Outcomes**	Test assessment (i.e., PDMS-2, 6-min walk test, and handgrip strength)			PEDI assessment		
	**Timepoints**	Baseline *n* = 84	Mid-int *n* = 83	End-of-int *n* = 81	Baseline *n* = 84	Mid-int *n* = 83	End-of-int *n* = 81
**Reasons for non-scheduled assessments**	Logistics (e.g., due to treatment procedures, ended treatment, treatment at another site)	8	8	3		2	
	The child could not cooperate with any health professionals at the time	1					
	Health condition/isolation	7	3	1			
	The parents did not want to participate due to the child’s health condition		2			2	
	The parents did not want to participate due to lack of mental surplus	1	4	2	1	5	2
	The child did not want to participate			1			
	The parents could not be reached		3	1		3	2
	Forgotten follow-up by the research team					1	
	Total of non-scheduled assessments	17 (20%)	20 (24%)	8 (10%)	1 (1%)	13 (16%)	4 (5%)

**Table 4:** Reasons for non-completion of the three outcomes of the test assessment (i.e., PDMS-2, 6-min walk test, and handgrip strength) at baseline (treatment initiation), mid-intervention (3 months), and end-of-intervention (6 months)

	**Outcomes**	PDMS-2			6-min walk test			Handgrip strength		
	**Timepoints**	Baseline *n* = 84	Mid-int *n* = 83	End-of-int *n* = 81	Baseline *n* = 84	Mid-int *n* = 83	End-of-int *n* = 81	Baseline *n* = 84	Mid-int *n* = 83	End-of-int *n* = 81
**Reasons for non-completed outcomes**	Pain/physical deficiencies (e.g., cannot walk/trouble with walking/health conditions/not feeling well)	14	1		31	10	9	9		
	No more energy/tiredness		1		2	2		1	1	
	Does not understand instructions				1	1	1	14	16	18
	Willingness due to temper/motivation/concentration	16	5	8	19	12	20	19	5	9
	Too long of a test battery		1		7	20	19	1	7	2
	Total non-completed outcomes	30 (36%)	8 (10%)	8 (10%)	60 (71%)	45 (54%)	49 (60%)	44 (52%)	29 (35%)	29 (36%)

### Adherence

**Fig. 2:**
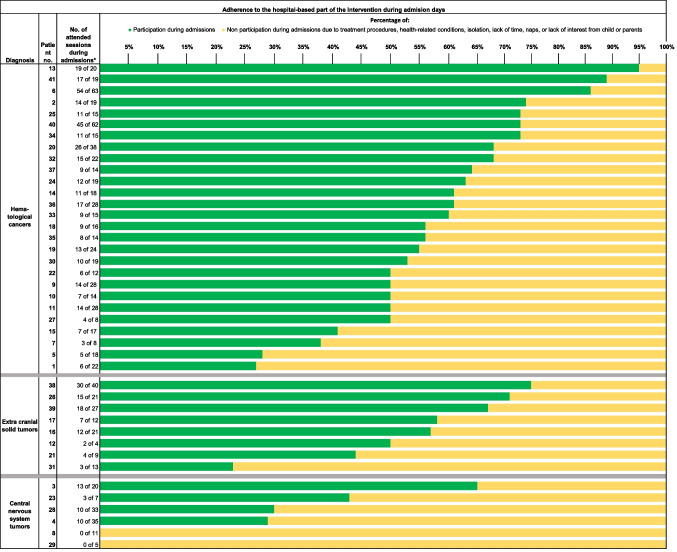
Adherence to the hospital-based part of the intervention during admission days in total numbers and percentage for each child in the intervention group. *Referring to the number of attended sessions of the number of admission days (excluding admission days on weekends, holidays, or days where the intervention was not offered) (i.e., patient no. 13 had 20 admission days during the 6-month intervention and attended the offered group or individual session on 19 of these days, corresponding to adherence of 95%)

During admissions, children allocated to the intervention group (*n* = 39; two children died before end-of-intervention) participated in a median of 11 [IQR 7; 15] hospital-based healthcare professional-led sessions during the 6 months of intervention, corresponding to a rate of 0.46 sessions per week. Adherence to the healthcare professional-led sessions at the hospital was analyzed by the number of attended sessions out of the number of possible sessions to attend during admission, resulting in a median adherence in the intervention group of 57% [IQR 50%; 68%]. Figure 2 shows an overview of each participant’s individual adherence in total numbers and percentage during admission days. Reasons for non-participation on admission days were treatment procedures (42%); health-related conditions (26%); naps (11%); lack of interest from child or parents (11%); lack of time (8%); and hospital procedure-required isolation (2%). A detailed overview of the week-to-week adherence to the hospital-based part of the intervention during admission days, outpatient clinic days, and non-hospital days for each child in the intervention group is presented in Supplemental file 3 (Fig. S1). Adherence to the home-based part of the intervention was not analyzed as only five families (13%) completed the logbook sufficiently.

### Adverse events

No severe adverse events were registered during the primary study period or during assessments. Two mild (and expected) adverse events were registered during the hospital-based structured active play sessions, both bruises due to the child falling in a play activity. One moderate adverse event was registered concerning outcome completion for a child in the control group. The child fell during testing and could not complete the remainder of the test session.

### Parent evaluation survey to inform feasibility

Thirty-five parents (88%) of 40 children in the intervention group (one child died before end-of-intervention) answered the survey. Among the respondents, 94% found the intervention relevant or very relevant for their child, citing reasons such as “it helps my child both physically and mentally” and “a fun break from everyday life at the hospital.” Similarly, 94% found the intervention relevant or very relevant for them as parents, citing reasons such as “a help to ensure movement” or “given us tools and inspiration for activities.” Regarding scheduling of the intervention, 54% of parents found the timing of the hospital-based group sessions unsuitable during admissions, and 66% found it was unsuitable on outpatient clinic days. When participation in group sessions was not possible, 94% reported that the option to have individual sessions at the ward was good or very good.

In terms of location, 49% of the parents found that the conduction of the intervention outside the ward was suitable, 37% were indifferent, and 14% did not answer. As for the intervention length, 83% thought the length of 6 months was appropriate, 14% thought the intervention should have been longer, and 3% thought it should have been shorter.

## Discussion

In the RePlay trial, we showed high acceptance and low attrition, which may be influenced by the perceived relevance of the study and the intervention’s design, initiation, and duration. The acceptance rate of 84% in our study can be regarded as high when compared to previous randomized controlled trials investigating the effectiveness of physical activity in children, including preschool-aged children. These studies report acceptance rates ranging from 45 to 90% with a weighted average of 61% [13, 26, 27, 32, 34, 35]. However, it is important to note that these prior studies reported acceptance rates aggregated across broad age ranges (1 to 19 years), and no results have been reported specifically for preschool-aged children. Consequently, direct comparison for this subgroup is not possible. Our study’s notably high acceptance rate may be attributable to the specific focus on children aged 1–5 years. Our findings show that the parents considered the intervention relevant for themselves and their child. This could be attributed to the early initiation of the intervention (within 2 weeks of treatment initiation), which places a focus on the child’s development—a focus that can be a concern for the parents early on [54]. Previous studies that reported high acceptance incorporated an early intervention initiated at diagnosis [13, 26, 35]. Moreover, studies employing an intervention where participation required no extra hospital visits have generally reported higher acceptance rates (mean 83% [range 77–90%]) [13, 26, 35] than studies where participation required extra hospital visits (mean 54% [range 45–66%]) [27, 32, 34]. Similarly, studies with a high demand of parents (i.e., extra hospital visits or daily parent-led exercises) for an extended duration (24–30 months) have higher attrition rates [13, 34], further indicating that a shorter intervention duration (e.g., during intensive treatment) might be important to support perceived relevance and limit dropouts.

The median adherence to the structured active play intervention at the hospital was 0.46 sessions/week, which may be influenced by the rigidity of the timeslots and restricted access to facilities. We showed a median adherence to the intervention during admission of 57%, which is lower than most reported adherence rates in previous physical activity intervention studies for children aged 1–18 years during cancer treatment (range 11–90%) [13, 26, 28–30, 32]. Adherence across studies appears to be influenced by intervention design. The lowest adherence (11%) was reported in a home-based, parent-led intervention with daily exercises [13]. In contrast, studies with higher adherence rates (> 68%; mean 79% (68–90%)) all conducted supervised in-hospital physical activity thrice weekly [26, 28–30, 32]. Compared to these studies, adherence in our study was relatively low despite also offering supervised sessions. In two of the previous studies, they only included children with leukemia [29, 30], and one study only included children with extracranial solid tumors [32]; in contrast, we include children with all diagnoses, and the children with central nervous system tumors had the lowest mean adherence to the intervention in our study. Overall, multiple factors might explain the varying adherence in our study. Treatment procedures (42%) and health-related conditions (26%) accounted for most registered reasons for non-participation in the intervention. Furthermore, the parent evaluation survey indicated that the timing of group sessions during admission was suboptimal for more than half of the families. These findings indicate that flexibility is essential. Additionally, we found in a qualitative observational study from RePlay that the children expressed joy of movement and a desire to play, indicating that the children themselves tolerated the intervention; however, we also found that their mood could quickly change, often associated with their physical well-being [55]. Although we designed the intervention with a structured approach that allows for adjustments to activities based on the child’s daily well-being and play preferences [43], the rigid design with scheduled timeslots and restricted access to facilities may hinder the accommodation of the child’s quickly changing mood. This was also present in 11% of the reasons for non-participation, being a lack of interest from the child or parents. Barriers such as treatment procedures, health-related conditions, naps, and the child’s changing interest with changing physical well-being may be avoided with greater flexibility and easier access to appropriate facilities.

Completing the primary outcome in our study was feasible at the end-of-intervention (80%); however, it posed certain challenges at baseline assessment (44%). The reasons for not completing the outcome at baseline were related to pain/physical deficiencies or unwillingness. Our outcome completion rates at baseline and end-of-intervention are comparable with previous studies showing completion rates at baseline of 51% and 53% and end-of-intervention of 89% and 93% of gross motor function outcomes in children diagnosed with cancer aged 1–8 years [13, 35]. Notably, these two previous studies had a broader age range and used the Movement Assessment Battery for Children (M-ABC and M-ABC-2), which only comprises eight tasks and is a less demanding test than the PDMS-2. In our study, the children aged ≥ 36 months had a lower completion rate at treatment initiation than the children aged < 36 months, which may be attributed to the test being more demanding with age. Overall, multiple factors may influence the test completion rates at treatment initiation, including treatment-related side effects, as seen affecting the completion in older children with a negative association between time since last chemotherapy and the child’s ability to complete the assessment [56]. Additionally, being at the hospital and having many procedures done within the first weeks of treatment may affect the children’s willingness to participate in further testing [57]. Lastly, the age of the children in our study may also play a role, as our findings showed that unwillingness was a reason for non-completion, which may be due to the long test battery and demanding tests. Collectively, these findings suggest that assessing gross motor function and physical capacity in the early stages of childhood cancer is complicated, albeit possible. Ensuring a short test battery with only the essential outcomes may minimize distress and amotivation, especially for preschool children.

The suitability of certain outcome measures for preschool children across the age group can be discussed. A previous study has evaluated the 6-min walk test in healthy children aged 3–11 years and found a completion rate of 39% in children aged 3–5 years [58], which aligns with another study where the lower completion rates for the 6-min walk test for healthy children under 5 years were attributed to non-physical reasons such as lack of attention [59]. The present study performed all three assessment outcomes (PDMS-2, 6-min walk test, and handgrip strength) within the same assessment, with PDMS-2 prioritized, which is reflected in the non-completion of the 6-min walk test, attributed to the overall length of the test battery and issues related to motivation or concentration. For handgrip strength, a prior study including healthy children aged 3–5 years found a completion rate of 98% [48]. Findings from our study show a completion rate of the handgrip strength test among the children ≥ 36 months of 80% at end-of-intervention, which is comparable to the study on healthy children. Notably, in our study, we also included children aged < 36 months. They showed low completion throughout the study in both the 6-min walk test and handgrip strength test with the main reason for non-completion being the inability to walk sufficiently and understand the instructions.

### Strengths and limitations

A key strength of this study is its exclusive focus on preschool-aged children—a population underrepresented in pediatric exercise oncology research. Thus, we have chosen to report the feasibility data in detail to inform future studies. The relatively large and sufficient sample size, high data completeness, and detailed reporting of feasibility are, hence, the advantages of the present study. Additionally, the high response rate to the parent evaluation survey provides further valuable insight into the practical aspects of the intervention, including its acceptance and adherence, from the parents’ perspectives. Despite these strengths, there are limitations that should be acknowledged. The single-site design may limit the generalizability of recruitment, adherence, and dropout patterns to other clinical settings, and the 16% who declined participation in the study may introduce selection bias. We did not have the ethical permission to collect data on the children who declined to participate. When looking at the percentage distribution of children between the diagnostic groups in our study (hematological cancers 66%, extracranial solid tumors 19%, and central nervous system tumors 15%), it differs from the percentage distribution seen in the overall childhood cancer population (i.e., hematological cancers 45%, extracranial solid tumors 30%, and central nervous system tumors 25%) [37]. Notably, hematological cancers, especially acute lymphoblastic leukemia, are predominantly prevalent in children aged 2–4 years [60], which may explain the larger distribution in this diagnostic group in our study.

### Implications for research and practice

The findings of this study offer important considerations for both practice and future research.

Multiple factors affect adherence to a rehabilitation intervention, especially for preschoolers during cancer treatment. Whether for research or practice, considering flexibility and proximity and ensuring easy access to facilities may be key when designing interventions or implementing initiatives.

To ensure high acceptability, it is paramount to have a pragmatic trial and intervention design that is perceived relevant for the patients and parents and is specifically tailored (e.g., home-based and/or hospital-based; structured physical activity or structured active play) to the needs and physical capabilities of the patients (e.g., preschoolers, children or adolescents) at the time of delivery (e.g., during intensive cancer treatment or maintenance treatment).

Physical assessment of preschoolers diagnosed with cancer is complex, particularly with objective outcome measures like the PDMS-2, which are dependent on the children’s active participation and willingness to do specific tasks. Nonetheless, the challenges associated with testing children with cancer during the early stages of treatment appear to be similar across different age groups. Therefore, further research should investigate different strategies for physical assessments in children with cancer.

When conducting physical assessments on preschoolers at any stage throughout the treatment, limiting the assessment battery and duration to the essential outcomes with measurement properties suitable for preschool children is advisable. If additional outcomes are needed, multiple assessment days may be the solution whenever possible. Furthermore, it is worth noting that the 6-min walk test and handgrip strength may not be suitable for children diagnosed with cancer < 36 months of age.

Lastly, finding solutions for physical activity and adherence monitoring for preschoolers at home is needed, as our findings showed that logbook registration at home for parents is not optimal.

## Conclusion

It is feasible to include and retain preschoolers and their parents in a rehabilitation intervention with structured active play during intensive cancer treatment. Although the assessment of gross motor function posed certain challenges, completing assessments at the end of the intervention was feasible, but future studies should consider the timing and duration of outcome assessments for this age group. The varying adherence to the intervention and the parent evaluation survey emphasize the importance of a flexible and easily accessible intervention, relevant for the patient group. Future studies should incorporate strategies to enhance adherence and minimize participation barriers.

## Supplementary Information

Below is the link to the electronic supplementary material.

Supplementary file1 (PDF 419 KB)

Supplementary file2 (PDF 196 KB)

Supplementary file3 (PDF 250 KB)

## Data Availability

The datasets generated and/or analyzed during the current study are not publicly available due to Danish and EU personal data legislation, but are available from the corresponding author upon reasonable request.

## Abbreviations

IQR: Interquartile range
PEDI: Pediatric Evaluation of Disability Inventory
PDMS-2: Pediatric Developmental Motor Scales – Second Edition
RePlay: Rehabilitation including structured active play for preschoolers with cancer
