# Supplementary material for: Evaluating the feasibility of a rehabilitation intervention including physical activity as structured active play for preschoolers diagnosed with cancer during the first 6 months of treatment—a study based on data from the RePlay trial
Source: Eur J Pediatr. 2025 Aug 6;184(8):533. doi: 10.1007/s00431-025-06350-y (PMC12325491; doi:10.1007/s00431-025-06350-y)
Supplement: Supplementary file 1 — Supplementary file1 (PDF 419 KB) [file 431_2025_6350_MOESM1_ESM.pdf]

Intervention evaluation questionnaire

The first 11 questions are regarding the structured active play intervention, RePlay, which you have participated in at the hospital.

1) According to you, how relevant is the RePlay study for your child diagnosed with cancer?

Very relevant	Relevant	Neither/nor	Less relevant	Not at all relevant

Briefly elaborate on your answer:

2) According to you, how relevant is the RePlay study for you as a parent of a child diagnosed with cancer?

Very relevant	Relevant	Neither/nor	Less relevant	Not at all relevant

Briefly elaborate on your answer:

3) Have you participated in the group structured active play sessions when you have been at the hospital?

Yes, always	Yes, mostly	Yes, sometimes	Rarely	Never

Why/why not?

4) Was the time of day for the group structured active play sessions suitable for you when you were admitted at the hospital? (Sessions for 1-2-year-olds were between 1-3 pm, and sessions for 3-5-year-olds were between 2-4 pm).

Yes	No

If no, what time of day would have been suitable?

Before 9 am	9-10 am	10-11 am	11-12 am	12-1 pm	1-2 pm	2-3 pm	3-4 pm	After 4 pm

Briefly elaborate on your answer:

5) Was the time of day for the group structured active play sessions suitable for you when you were visiting the outpatient clinic at the hospital? (Sessions for 1-2-year-olds were between 1-3 pm, and sessions for 3-5-year-olds were between 2-4 pm).

Yes	No

If no, what time of day would have been suitable?

Before 9 am	9-10 am	10-11 am	11-12 am	12-1 pm	1-2 pm	2-3 pm	3-4 pm	After 4 pm

Briefly elaborate on your answer:

6) **When you did not have the opportunity to participate in the group structured active play sessions, how did you then find the possibility of having individual sessions in the hospital room?**

Very good	Good	Neither/nor	Bad	Very bad

Briefly elaborate on your answer:

7) **Has the physiotherapy room been good as a facility for structured active play?**

Yes	No

Why/why not?

8) **How has it been having structured active play sessions outside the ward?**

Good	Neither/nor	Bad

Briefly elaborate on your answer:

9) Overall, has your child been motorically challenged in the structured active play sessions?

Very much so	Much so	Appropriately	To a lesser degree	Not at all

If you answered, “Very much so” or “Much so”, is this positive or negative?

Positive	Negative

Briefly elaborate on your answer:

10) Were you inspired by your participation in the RePlay Study to physically activate your child?

Very much so	Much so	Appropriately	To a lesser degree	Not at all

Briefly elaborate on your answer:

11) Do you use the activities from the structured active play sessions at home?

Very often	Often	Occasionally	Rarely	Never

Briefly elaborate on your answer:

The next 9 questions are regarding the inspirational material/play folder that you got through your participation in RePlay.

1) **Do you understand the descriptions of the play activities in the play folder?**

Yes, fully	Yes, partially	No

2) **Do you understand the colors of the play activities in the play folder?**

Yes, fully	Yes, partially	No

3) **How useful were the drawings in regard to understanding the activities?**

Very useful	Useful	Appropriate	Less useful	Not useful

Briefly elaborate on your answer:

4) **Have you used the play folder?**

Very much so	Much so	Occasionally	To a lesser degree	Not at all

Why/why not?

5) **How often have you used the play folder?**

Daily	Weekly	Monthly	Rare than monthly	Never

6) In what way have you used the play folder?

7) **To what degree have you involved your child in the use of the play folder?**

Very much so	Much so	Occasionally	To a lesser degree	Not at all

If you have involved your child to occasionally or less, why so?

	My child is too young
	My child does not show interest
	The material is not useful with the child
	We just have not done it
	Other

8) Have you completed the logbook?

Yes, completely	Yes, partially	No

Briefly elaborate on your answer:

9) **How often have you done structured active play at home?**

Daily	Weekly	Monthly	Rare than monthly	Never

Briefly elaborate on your answer:

The last 5 questions are regarding extra information and your overall rating of the RePlay study.

1) **Has your child been referred to physiotherapy while participating in the RePlay study?**

Yes	No

If yes, why so?

____

If yes, how often and for how long?

2) **Has your child been going to the SIV-house while participating in the RePlay study?**

Yes	No

How often?

3) To what degree has the RePlay study fulfilled your expectations?

Very much so	Much so	Appropriately	To a lesser degree	Not at all

Briefly elaborate on your answer:

4) How would you rate the length of the intervention?

It should be shorter	It is appropriate	It should be longer

Briefly elaborate on your answer:

5) On a scale from 1-10, what is your overall opinion on the RePlay intervention with structured active play?

Very bad Very good

1	2	3	4	5	6	7	8	9	10
