# Supplementary material for: Evaluating the feasibility of a rehabilitation intervention including physical activity as structured active play for preschoolers diagnosed with cancer during the first 6 months of treatment—a study based on data from the RePlay trial
Source: Eur J Pediatr. 2025 Aug 6;184(8):533. doi: 10.1007/s00431-025-06350-y (PMC12325491; doi:10.1007/s00431-025-06350-y)
Supplement: Supplementary file 2 — Supplementary file2 (PDF 196 KB) [file 431_2025_6350_MOESM2_ESM.pdf]

Supplemental Table S1: The sub-analysis of the outcome completion on age (<36 months and ≥36 months), sex (boys and girls), diagnostic groups (hematological cancers (Hem), extra cranial solid tumors (Solid), central nervous system tumors (CNS)), and trial group (intervention (Int.) and usual care (UC)).

	Baseline **n=84**									Mid-intervention **n=83**									End-of-intervention **n=81**								
	Age <36 months **n=33**	Age ≥36 months **n=51**	Male **n=42**	Female **n=42**	Hem **n=55**	Solid **n=16**	CNS **n=13**	UC group **n=43**	Int. group **n=41**	Age <36 months **n=32**	Age ≥36 months **n=51**	Male **n=42**	Female **n=41**	Hem **n=54**	Solid **n=16**	CNS **n=13**	UC group **n=42**	Int. group **n=41**	Age <36 months **n=31**	Age ≥36 months **n=50**	Male **n=42**	Female **n=39**	Hem **n=53**	Solid **n=16**	CNS **n=13**	UC group **n=41**	Int. group **n=40**
**Fully completed PDMS-2**	17 (52%)	20 (39%)	14 (33%)	23 (55%)	20 (36%)	9 (56%)	8 (62%)	18 (42%)	19 (46%)	20 (63%)	35 (69%)	28 (67%)	27 (66%)	43 (80%)	7 (44%)	5 (39%)	29 (69%)	26 (63%)	23 (74%)	42 (84%)	31 (74%)	34 (87%)	46 (87%)	10 (63%)	9 (69%)	34 (83%)	31 (78%)
**Partially completed PDMS-2**	2 (6%)	13 (25%)	9 (21%)	6 (14%)	12 (22%)	1 (6%)	2 (15%)	6 (14%)	9 (22%)	3 (9%)	4 (8%)	5 (12%)	2 (5%)	5 (9%)	2 (13%)	0 (0%)	5 (12%)	2 (5%)	3 (10%)	2 (4%)	4 (10%)	1 (3%)	3 (6%)	1 (6%)	1 (8%)	2 (5%)	3 (8%)
**Handgrip strength**	0 (0%)	23 (45%)	10 (24%)	13 (31%)	15 (27%)	2 (13%)	5 (38%)	13 (30%)	10 (24%)	2 (6%)	32 (63%)	18 (43%)	16 (39%)	29 (54%)	3 (19%)	2 (15%)	19 (45%)	15 (37%)	4 (13%)	40 (80%)	23 (55%)	21 (54%)	36 (68%)	4 (25%)	4 (31%)	23 (56%)	21 (53%)
**2 minute walk test**	1 (3%)	8 (16%)	2 (5%)	7 (17%)	4 (7%)	1 (6%)	4 (31%)	4 (9%)	5 (12%)	5 (16%)	16 (31%)	12 (29%)	9 (22%)	17 (31%)	2 (13%)	2 (15%)	10 (24%)	11 (27%)	5 (16%)	24 (48%)	13 (31%)	16 (41%)	22 (42%)	4 (25%)	3 (23%)	16 (39%)	13 (33%)
**6 minute walk test**	1 (3%)	6 (12%)	1 (2%)	5 (12%)	3 (5%)	1 (6%)	2 (15%)	4 (9%)	3 (7%)	4 (13%)	14 (27%)	10 (24%)	8 (20%)	14 (26%)	2 (13%)	2 (15%)	10 (24%)	8 (20%)	5 (16%)	19 (38%)	11 (26%)	13 (33%)	17 (32%)	4 (25%)	2 (15%)	15 (37%)	9 (23%)
**Completion of full test-session (i.e., PDMS-2, 6MWT, HG)**	0 (0%)	4 (8%)	1 (2%)	3 (7%)	3 (5%)	0 (0%)	1 (8%)	2 (5%)	2 (5%)	2 (6%)	11 (22%)	7 (17%)	6 (15%)	11 (20%)	1 (6%)	1 (8%)	6 (14%)	7 (17%)	3 (10%)	19 (38%)	10 (24%)	12 (31%)	18 (34%)	2 (13%)	2 (15%)	13 (32%)	9 (23%)

Among children <36 months of age, none completed the handgrip strength test at baseline, two (6%) at mid-intervention, and four (13%) at end-of-intervention. The primary reason for non-completion was difficulty understanding the instructions for the test. For the 6-minute walk test, one child (3%) in this age group completed baseline, four (13%) at mid-intervention, and five (16%) at end-of-intervention. Reasons for non-completion included difficulty walking, the length of the test battery, and unwillingness to participate. Among children aged ≥36 months, 23 (45%) completed the handgrip strength at baseline, 32 (63%) at mid-intervention, and 40 (80%) at end-of-intervention. For the 6-minute walk test, six (12%) completed the test at baseline, 14 (27%) mid-intervention, 19 (38%) at end-of-intervention. Children diagnosed with hematological cancers had a lower completion rate of the PDMS-2 at baseline (36%) compared to children in the other diagnostic groups (56% and 57%). However, this trend reversed at mid-intervention, with 80% of children in the hematological cancers group completing the full PDMS-2, compared to 39% and 44% in the other groups. By the end of the intervention, completion rates were 87% for the hematological cancers group and 63% and 69% for the two other groups. The large discrepancies in the hematological cancers group between completion rates at baseline and end-of-intervention can be explained by the fact that this group accounts for 75% of the logistical issues with scheduling due to treatment procedures and 57% of the scheduling failures due to health conditions at baseline. Furthermore, the children in the hematological cancers group account for 81% of the non-completed PDMS-2 tests due to willingness, which can be explained by the fact that this group of children is already receiving Dexamethasone within the first month of treatment, affecting their mood. The lower completion rate at mid-intervention in the extra cranial solid tumors group and the central nervous system tumors group can be found in these groups accounting for 70% of the non-scheduled tests, mainly due to surgery, treatment with radiation at another site, or child's health condition (i.e., palliative care, paralysis).
