# Supplementary material for: Evaluating the feasibility of a rehabilitation intervention including physical activity as structured active play for preschoolers diagnosed with cancer during the first 6 months of treatment—a study based on data from the RePlay trial
Source: Eur J Pediatr. 2025 Aug 6;184(8):533. doi: 10.1007/s00431-025-06350-y (PMC12325491; doi:10.1007/s00431-025-06350-y)

Supplemental Figure S1: The figure shows: 1) a detailed overview of the week-to-week adherence to the hospital-based part of the intervention during admission days, outpatient clinic days, and non-hospital days for each child in the intervention group; 2) a detailed overview of reasons for non-participation during admission days presented in color codes with descriptions.

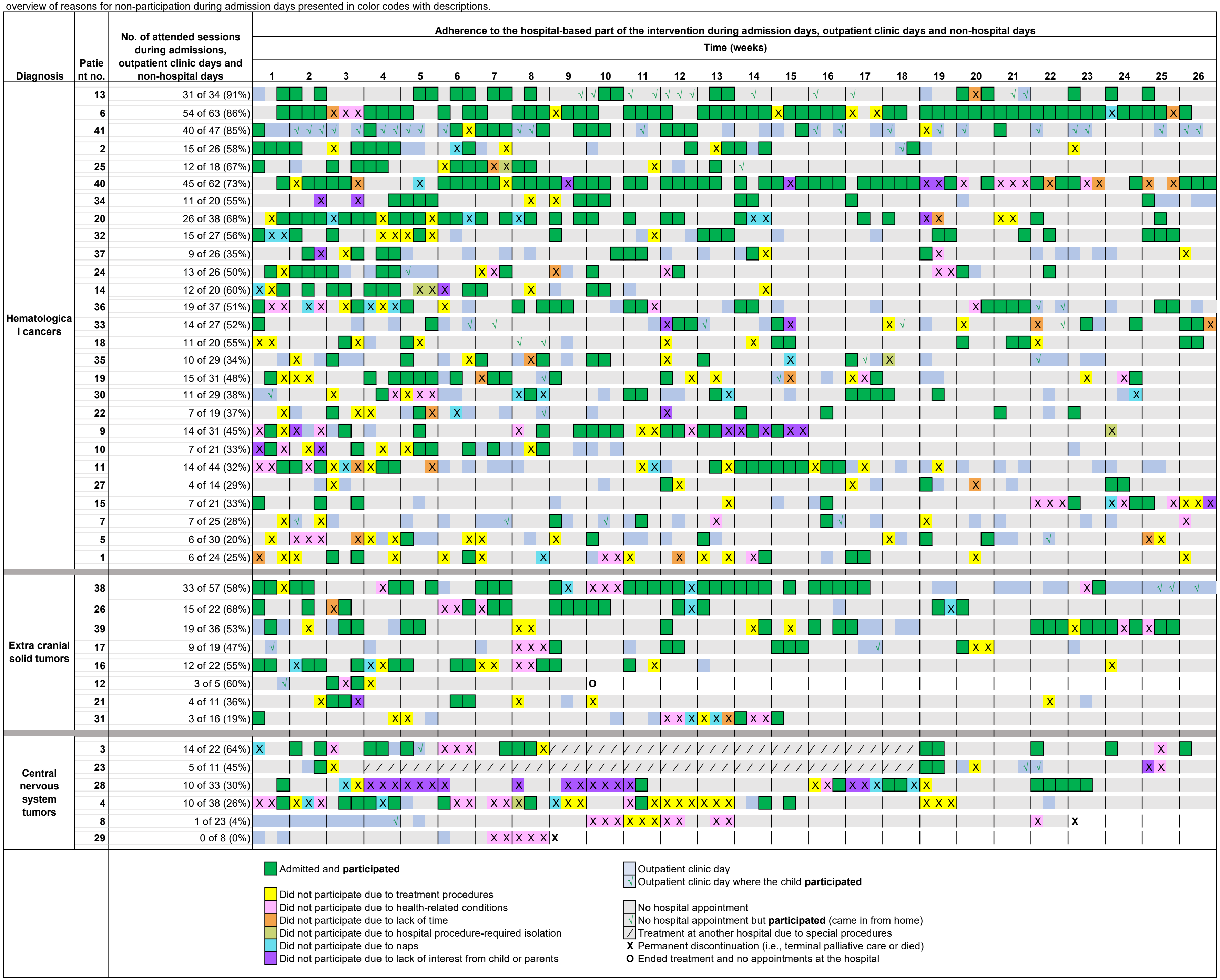

Supplement: Supplementary file 3 — Supplementary file3 (PDF 250 KB) [file 431_2025_6350_MOESM3_ESM.pdf]
